# Balance and fragmentation in societies with homophily and social balance

**DOI:** 10.1038/s41598-021-96065-5

**Published:** 2021-08-25

**Authors:** Tuan M. Pham, Andrew C. Alexander, Jan Korbel, Rudolf Hanel, Stefan Thurner

**Affiliations:** 1 Section for the Science of Complex Systems, CeMSIIS, Medical University of Vienna, Spitalgasse 23, 1090 Vienna, Austria; 2 Complexity Science Hub, Vienna Josefstädterstrasse 39, 1090 Vienna, Austria; 3 Department of Mathematics, Princeton University, Princeton, NJ 08544 USA; 4 Santa Fe Institute, 1399 Hyde Park Road, Santa Fe, NM 87501 USA

**Keywords:** Statistical physics, thermodynamics and nonlinear dynamics, Applied mathematics

## Abstract

Recent attempts to understand the origin of social fragmentation on the basis of spin models include terms accounting for two social phenomena: homophily—the tendency for people with similar opinions to establish positive relations—and social balance—the tendency for people to establish balanced triadic relations. Spins represent attribute vectors that encode *G* different opinions of individuals whose social interactions can be positive or negative. Here we present a co-evolutionary Hamiltonian model of societies where people minimise their individual social stresses. We show that societies always reach stationary, balanced, and *fragmented* states, if—in addition to homophily—individuals take into account a significant fraction, *q*, of their triadic relations. Above a critical value, $q_c$, balanced and fragmented states exist for any number of opinions.

## Introduction

The concept of so-called *filter bubbles* captures the fragmentation of society into isolated groups of people who trust each other, but clearly distinguish themselves from “other”. Opinions tend to align within groups and diverge between them. Interest in this process of social disintegration, started by Durkheim^1^, has experienced a recent boost, fuelled by the availability of modern communication technologies. The extent to which societies fragment depends largely on the interplay of two basic mechanisms that drive social interactions: *homophily* and *structural balance*. Homophily is the “principle” that “similarity breeds connection”^2^. In particular, for those individuals who can be characterised by some social traits, such as opinions on a range of issues, homophily appears as the tendency of like-minded individuals to become friends^3^. The concept of *structural balance*, first described by Heider^4^, can be translated into a tendency of unbalanced triads to become balanced over time. A triad of individuals is *balanced* if all three individuals are mutual friends (friend of my friend is my friend) or if two friends have a mutual enemy (enemy of my enemy is my friend). Structural balance has been investigated by social scientists for a long time^5–7^ and, more recently, by physicists and network scientists^8–24^. Recent contributions study the dynamics on balanced networks^25–27^, the co-evolution of opinions and signed networks^28–40^, and generalized measures of structural balance^41, 42^. For an overview, see^43,44^. A general survey of statistical physics methods applied to opinion dynamics is found in^45,46^.

Previous works studying social fragmentation under the joint effects of homophily and social balance have been in only partial agreement with Heider’s theory. For example, in an attribute-based local triad dynamics model (ABLTD)^47^ each agent has binary opinions on *G* attributes. If two agents agree on more attributes than they disagree on, they become friends (positive link). Agents tend to change their attributes to reduce stress in unbalanced triads. The paper showed that given a system of *N* agents, as $N \rightarrow \infty$, the so-called “paradise state”, where all agents are friends of each other, is never reached unless the number of attributes *G* scales as $O(N^\gamma )$, with $\gamma \ge 2$. Instead, the society remains in a stationary *unbalanced* state with an equal number of balanced and unbalanced triads. Realistic social networks, where *N* is typically large and *G* remains relatively small, are hence expected to be unlikely to reach social balance, let alone the paradise state. This statement is to some extent contrary to empirical findings that societies are balanced to a high degree; see e.g. recent work on large scale networks^16,17^.

In another, so-called *global social stress Hamiltonian* framework^12,40,48–51^, the opinion of individual *i* is denoted by $s_i$ and the relation between *i* and *j* by $J_{ij}$ (positive or negative). Defining a social stress, *H*, as the sum of a homophily-related term, $- \sum _{(i,j)} J_{ij} s_i s_j$ and a term reflecting social balance, $-\sum _{(i,j,k)} J_{ij}J_{jk}J_{ki}$, it can be shown that societies, where social balance is present, necessarily become fragmented at some critical level of interconnectedness^40^. This result, however, is restricted to the case where the reduction of *H* can be realised by either an opinion update or a flip of link’s sign with the latter happening to be independent from the former. Social relations, which are subject to a homophily effect, essentially depend on the agents’ similarity in opinions, and hence necessarily evolve as opinions are updated.

In this paper, motivated by the lack of a consistent theory of balance and fragmentation in societies of agents with multidimensional opinions and homophilic interactions, we propose an *individual-stress-based* model that takes into account the homophily effect between adjacent individuals and structural balance within a time-varying local neighborhood. The latter consists of the subset of the most relevant triads to an individual at a given moment in time, i.e. those that involve the relationships that are currently in their field of attention when considering their social stress. The ratio of relevant triads to the total number of triads the individuals belong to determines whether society fragments or remains cohesive. With the help of simulations on a regular network, we show that there exists a critical size of the local neighborhood above which society fragments, yet stays balanced. We discuss the relation of the presented model to both, the ABLDT model^47^ and the social stress Hamiltonian approach^40^.

## Results

### Local social stress model

Consider a society of *N* individuals. Each individual *i* has binary opinions on *G* issues, characterized by an attribute vector, $\mathbf{A} _i = \{a_i^{\ell }\}$, where $a_i^{\ell } \in \{-1,+1\}$; $\ell \in 1,\dots ,G$. Further, *i* has relations to $k_i$ other individuals in a social network. Network topology does not change over time. Following^47^, the relation between two agents *i* and *j* is determined by the sign of their distance in attribute space: $J_{ij} = \mathrm{sign}(\mathbf{A} _i \cdot \mathbf{A} _j)$, where the dot denotes the scalar product. $J_{ij} = 1$ indicates friendship, $J_{ij}=-1$ enmity. Each agent *i* has a social stress level, $H^{(i)}$, defined as

1 $$\begin{aligned} H^{(i)}(\mathbf{A} )= - \frac{1}{G}\, \sum _{j} J_{ij} \mathbf{A} _i \cdot \mathbf{A} _j -\sum _{(j,k)_{Q_{i}}} J_{ij}J_{jk}J_{ki} \, . \end{aligned}$$

The first sum extends over all $k_i$ neighbours of *i*, while the second is restricted to $Q_i$ out of $N^{\Delta }_i$ triads that node *i* belongs to (by definition, $N^{\Delta }_i \equiv c_i k_i(k_i-1)/2$, where $c_i$ is the local clustering coefficient). The relevance of this term in the model dynamics is discussed in the Supplemental Material. The notation $(j,k)_{Q_i}$ means to sum over all pairs of *j* and *k* which, together with *i*, form the $Q_i$ triads. These are chosen at each step of the dynamics (see below). $Q_i$ represents the number of triads *i* would like to have socially balanced—*i* ’s relevant neighborhood to the current stress-calculation. The existence of this neighborhood limits the extent to which the social network can change at any given update. Specifically, those edges that do not belong to the $Q_i$ triads, will *not* be updated. The idea behind this is that such links preserve a memory of *i*’s relationships (at a previous time) with those who are currently not in the field of attention of *i*. As such, these links do not change instantaneously as *i* updates his opinion. For example, you may have an outdated relation to an old school friend until you two meet again at a class reunion and find out you still like each other or perhaps not. The factor 1/*G* ensures that contributions from any link towards $H^{(i)}$ do not diverge in the limit $G \rightarrow \infty$. Assuming agents try to minimize their individual social stress over time, we implement the following dynamics:

1. *Initialize*. Each node is assigned an opinion vector, $\mathbf{A} _i$, whose components are randomly chosen to be 1 or $-1$ with equal probability. Every node has the same degree, $k_i = K$, and is connected to its neighbours in a regular way, forming the ring topology. The topology is fixed over time. For any pair of connected agents, *i* and *j*, we set $J_{ij} = \mathrm{sign}(\mathbf{A} _i \cdot \mathbf{A} _j)$.
2. *Update*. (i) Pick a node *i* randomly and choose $Q_i$ of its triads, also randomly. Compute $H^{(i)}$. In the current state its value is $\mathcal {H}$. (ii) Flip one of *i*’s attributes at random. Let $\tilde{\mathbf{A }}_i$ be its new opinion vector. For each of the chosen triads, the weights of the two links adjacent to *i* are recomputed as $\tilde{J}_{ij} = {\mathrm{sign}}(\tilde{\mathbf{A }}_i \cdot \mathbf{A} _j)$. $\tilde{J}$ is the new matrix. Compute the new stress $\tilde{\mathcal {H}}$ using $\tilde{J}$. The change in stress is $\Delta H^{(i)} \equiv \tilde{\mathcal {H}}-\mathcal {H}$.
  (iii) Update the system $\mathbf{A} _i \rightarrow \tilde{\mathbf{A }}_i$ and $J_{ij} \rightarrow \tilde{J}_{ij}$ with probability, $\min \left\{ e^{ - \Delta H^{(i)}},1\right\}$, otherwise leave it unchanged. This stochastic rule means that agents are not always rational and might choose to increase their stress.
3. Continue with the next update of opinions and links by returning to step 2.

**Figure 1:**
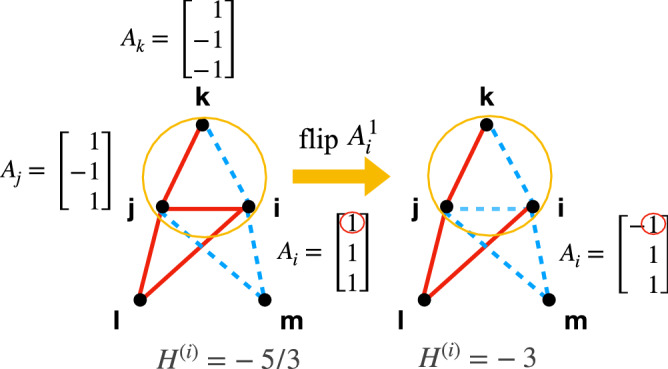
Co-evolutionary interplay of opinions and links. Red (blue) links denote positive (negative) relationships. Among the three triads in the graph, the chosen one, (*i*, *j*, *k*) is circled. We consider the case $Q = 1$. As agent *i* flips one of its attributes, $A_i^1$, this triad becomes balanced and *i* decreases its individual stress from $-5/3$ to $-3$. The opinion vectors of *l* and *m* are $\mathbf{A} _l = \mathbf{A} _i$ and $\mathbf{A} _m = - \mathbf{A} _i$, respectively (not shown in the figure). Links that do not belong to the three depicted triangles are not shown in the figure. Note that the full network is a ring.

Figure 1 illustrates an update where by changing one opinion, agent *i* becomes an enemy of *j*, but the chosen triad, (*ijk*), becomes balanced. If $Q=1$, this decreases *i*’s social stress from $H^{(i)} = -5/3$ to $H^{(i)} = -3$. If more triads are chosen, $Q>1$, this flip leads to a stress increase and is less likely accepted.

The change in stress for agent *i*, $\Delta H^{(i)}$, given that attribute $a_i^{\ell _*}$ flips, can be written as

2 $$\begin{aligned} \Delta H^{(i)}= \frac{2}{G}\, \sum _{(j | \tilde{J}_{ij} = J_{ij})} J_{ij} a_i^{\ell _*} a_j^{\ell _*} + \sum _{(j,k)_Q} \Delta _{jk} \, , \end{aligned}$$

where $j | \tilde{J}_{ij} = J_{ij}$ means to sum over those *j* (neighbours of *i*) for whom the sign of the edge $J_{ij}$ remains unchanged and $\Delta _{jk} = \big [J_{ij}J_{ki} - \tilde{J}_{ij}\tilde{J}_{ki}\big ] J_{jk}$. Obviously, $\Delta _{jk} \in \{-2, 0, 2\}$. According to the dynamical rule, the maximum number of links that may change their signs due to an opinion update depends on *Q*. Since most links are kept frozen for a small *Q*, the dynamics is mainly driven by the first term that makes friends more similar while enemies more dissimilar. Note the similarity to the Hebbian rule^34,52^. Because of the random assignment of the opinions at the start, there are approximately as many balanced as unbalanced triads in the stationary state. For large *Q*, the change in the energy related to social balance can be very large, increasing the chance to reach a balanced state without unbalanced triads. In this state, all individuals are expected to have a minimum amount of social stress.

### Order parameter

To measure the level of social balance within a society, we define an order parameter, *f*, as the difference between the proportions of balanced and unbalanced triangles:

3 $$\begin{aligned} f = \frac{n_{+} - n_{-}}{n_{+} + n_{-}}, \end{aligned}$$

where $n_{+}$ and $n_{-}$ are the number of balanced and unbalanced triangles, respectively. $f= 1$ means that all triangles are balanced, $f < 1$ signals the presence of unbalanced triangles. A network is called *balanced*^53^ if and only if all cycles (including triangles as cycles of length 3) contain only an even number of negative edges. In our study, rather than following this strict mathematical definition of balanced graphs, we propose to call a society *balanced*, if all of its constituent triads are balanced. Fully-connected balanced networks are two-clusterable, i.e., they can be partitioned into two *clusters of friends*, within which all links are positive and between which links are exclusively negative^53^. For these networks, $f = 1$ is a necessary and sufficient condition for such a bipartition as they having all triads balanced is equivalent to having cycles of any length balanced. However, as we would only call a network *fragmented* (*k*-clusterable in the signed network literature), if it can be decomposed into $k \ge 2$ clusters of friends, it is worth to noting that balanced states are generally different from fragmented ones in incomplete networks. This is because, for an incomplete network, all triangles may be balanced while leaving some cycles of larger lengths unbalanced. Therefore, being *triad-wise* balanced ($f = 1$) is a necessary, but not sufficient condition for being fragmented (*k*-clusterable)^53^.

### Results

**Figure 2:**
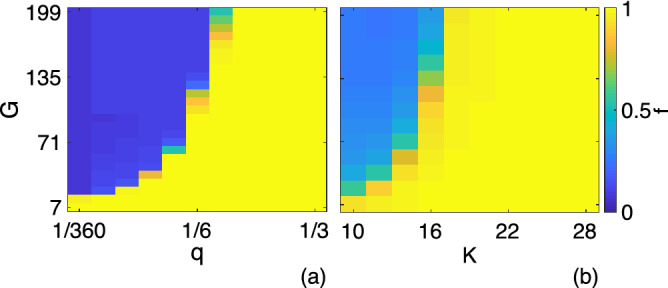
Order parameter, *f*, **(a)** as a function of *q* and *G* for $K = 32$, and **(b)** as a function of *K* and *G* with $q = 1/3$. Results are averaged over 100 runs on a regular ring network with $N=400$.

We first run the simulation on a regular ring network for $N=400$, where every node has a degree of $K = 32$ neighbors. Figure 2a shows a phase diagram of the order parameter, *f*, which indicates a transition from an unbalanced to a balanced society. For any given *G*, this transition occurs as $q \equiv Q/N^\Delta$ passes a threshold $q_c$. The existence of a critical $q_c$ demonstrates the importance of Heider’s balance term in driving a society towards social balance: if a sufficiently large number of triangles is taken into account, society becomes balanced. For a wide range of *G*, $q > 1/5$ clearly suffices to be in the balanced phase. Also, $q_c$ increases with growing *G*, indicating that when more issues become relevant for homophily, the chance for achieving balance lowers. This can be understood as follows. The probability that a link incident with *i* switches its sign if $a_i^{\ell _*}$ flips, is proportional to $1/\sqrt{G}$, as $G \rightarrow \infty$ (see the Supplemental Material for the derivation of this asymptotic formula). Therefore, links are less likely to change as *G* increases, making it harder for the dynamics to happen and the society to become balanced. Note that the situation resembles non-equilibrium in the sense that the quasi-stationary *unbalanced* states in the cohesive phase, due to fluctuations in finite-sized systems, eventually become highly *balanced* after a very long time. As the presented model is stochastic, these final states are not necessarily frozen (absorbing). This means that a small number of unbalanced triads still fluctuates over time. The transition is presumably first-order, as a region of bi-stability is numerically observed where the order parameter can be $f \sim 0$ or $f \sim 1$; see Supplemental Material for examples of the transition at the critical value of *Q*. Since the total number of triads per agent, $N^{\Delta }$, grows with *K*, *Q* must also grow with *K* as long as $q = Q/N^{\Delta }$ fixed. Therefore, the balanced phase is expected to be reached if the network degree exceeds a critical value, $K_c$. We verify this hypothesis in Fig. 2b for $q=1/3$. Interestingly, the transition becomes sharper at higher *K*.

**Figure 3:**
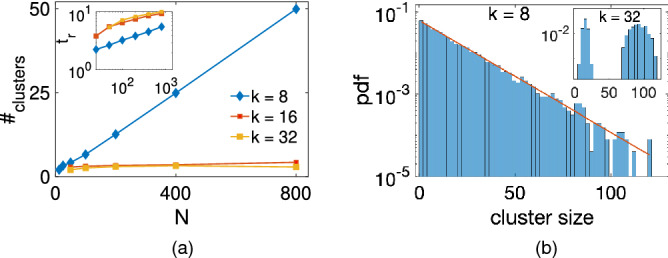
**(a)** Average number of clusters and average time to reach the balanced states, $t_r$, with $f = 1$ (inset), as a function of *N* for $K = 8, 16, 32$. Results are averaged over 1000 runs on a regular ring network for $N=12, 25, 50, 100, 200, 400$ and over 100 runs for $N = 800$ with $G = 9$ and $Q = 16$. **(b)** Probability density function of cluster sizes for $K = 8$ (main plot) and $K = 32$ (inset). The red line indicates a linear fit on a semi-log scale. Here $N=200$, $G = 9$ and $Q = 16$. One observes an exponential (bimodal) distribution for $K = 8$ ($K = 32$) networks, respectively.

We next study the scaling behaviour of the time to reach a balanced steady state and the number of clusters of friends in this state with the system size. The latter is investigated in order to check whether the balanced states are also fragmented. For $N = 50,100,200,400,800$, the results in Fig. 3a demonstrate that the number of clusters grows with *N* for $K = 8$, but remains small for higher degrees $K =16, 32$. In both cases, the fragmented state tends to persist also in the thermodynamic limit as long as the average number of clusters are always larger or equal to two. Further, the time to reach balance grows as $t_r \propto N^\alpha$, with $\alpha < 1$ for $K = 8$, and $t_r$ appears to be a convex function of *N* for $K = 16, 32$, suggesting that it may saturate at some point, see inset in Fig. 3a. This means that the balanced phase should always be reached even though the time it takes may be quite long for very large systems. It would also be interesting to understand the temporal evolution of the number of clusters and to establish whether this number can converge to two at long times for $K = O(N)$. Finally, we find that the distribution of cluster sizes follows an exponential for networks with $K =8$, but shows a bimodal distribution for those with $K = 32$, see inset in Fig. 3b. We can intuitively understand this observation as follows: A balanced network is expected to have a cluster statistics similar to that of an unsigned graph that can be obtained from it by removing all negative edges. For small *K*, this unsigned network has an expected number of connected components proportional to *N* (Fig. 3a main). Therefore, it must have an average degree, $\tilde{K}$, of positive edges that is below the percolation threshold for the emergence of giant component. That below this threshold unsigned networks, with high probability, exhibit an exponential distribution of component sizes, in full consistency with the distribution observed in the original balanced network. This situation changes as the degree, *K*, of the original network increases. In the limit of $K \rightarrow N -1$, only two clusters of friends can emerge. While in general these two clusters can have different sizes, in the most probable configuration they are of almost equal size. This gives rise to a single peak at *N*/2 in the cluster distribution of fully-connected networks. In the intermediate range of *K*, a bimodal distribution necessarily occurs as a crossover between the exponential and the singly-peaked ones.

#### Limit of small Q

In the limit $Q = 1$ and $G \rightarrow \infty$, the society is expected to reach an unbalanced stationary state, where the order parameter *f* is close to zero. We show this by a mean-field approach for fully-connected networks; see Supplemental Material. Here we assume that the two links of a chosen triad are not likely to be flipped simultaneously, as $G \rightarrow \infty$. Instead, only one of them would be flipped at every update. We then derive a set of rate equations for triads of different types whose steady state solution is $f^{(\rm{st})} = 0$ and $\rho _+^{(\rm{st})} = 1/2$, where $\rho _+$ denotes the fraction of positive links. These values of *f* and $\rho _+$ are the same as those obtained in the ABLTD model^47^ for $G = O(N^\gamma )$, $N\rightarrow \infty$ with $\gamma < 2$. Note, however, the models show different results for $G = O(N^\gamma )$, $N\rightarrow \infty$ with $\gamma \ge 2$. For a discussion on the similarities and differences of the models, see Supplemental Material.

#### Limit of large Q

Another interesting limit is when $Q \rightarrow N^{\Delta }$. In this case, one can compare the model with the Hamiltonian approach used in^40^, in which the contribution of *all* $N \times N^{\Delta }/3$ triangles, weighted by a coupling *g*, is taken into account:

4 $$\begin{aligned} \bar{H} \equiv - \frac{1}{2G}\, \sum _{(i, j)} J_{ij}\, \mathbf{A} _i \cdot \mathbf{A} _j - g \sum _{(i, j, k)} J_{ij}J_{jk}J_{ki}. \end{aligned}$$

**Figure 4:**
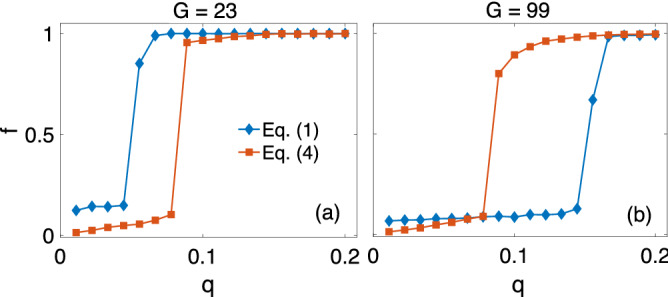
Limit of large Q. Comparison of the presented model in Eq. (1) and the one in Eq. (4) that resembles^40^. The coupling, *g*, is chosen to be $g = q\equiv Q/N^{\Delta }$, where *Q* is the number of actually updated triads; $N^{\Delta }$ is the total number of triads. Results are averaged over 100 independent runs for $N=100$, $K = 32$, $N^{\Delta } = 360$, and $G = 23$ **(a)**; $G = 99$ **(b).**

Here the first sum extends over all connected pairs, the second over all triangles. In Eq. (4), in contrast to the model presented here, $J_{ij}$ are random dynamical variables that co-evolve with, but are not strictly determined by the opinion vectors. The detailed updating procedure of^40^, which aims at minimizing $\bar{H}$, is described in the Supplemental Material. Despite the differences in the concrete update dynamics, for a large enough $Q \ge Q_{MF}$, the two models are expected to yield similar results if *g* is related to *Q* by $g = \alpha Q / N^{\Delta }$, for some constant $\alpha$. Here the main idea is that for sufficiently large *Q*, individuals’ actions have a similar outcome regardless of their knowledge of the total stress $\bar{H}$ in the society. Figure 4 shows the comparison for $\alpha = 1$. The curve of the presented model indeed crosses that of the model given by Eq. (4) at $q_1 \ge Q_{MF}/ N^{\Delta } \simeq 0.133$ for $G = 23$ in (a), and at $q_2 \ge 1/6$, for $G = 99$ in (b), where the coupling, *g*, is chosen to be equal to $q_1$ in (a) and $q_2$ in (b), respectively.

## Discussion

We showed that under the simultaneous effects of homophily and structural balance society can achieve structurally balanced states if individuals’ opinions co-evolve with their social links so as to minimize their *individual* social stress. The parameter *G* controls the dimension of the opinion vectors relevant for homophily, and $Q_i$ specifies how many triangles individuals actually consider for their local social balance. The interplay between homophily and structural balance results in a nontrivial phase diagram showing an abrupt change in patterns of social structure. We find two regimes: fragmentation and cohesion. In the former, society is fragmented into locally cooperative clusters of agents who are linked positively within and negatively between clusters. In the latter, globally percolating cooperation is realized by the existence of a large connected component of positively linked agents. The transition between the regimes is numerically observed at a critical fraction of the considered triangles, $q_c$, illustrating the main message of the paper: The more people try to balance their social neighborhoods, the more likely society is to become fragmented. Because of the relation between $Q_i$ and *K*, this message is robust with respect to the change of the network connectivity; for a fixed value of *q*, we see that the higher the degree, the more likely the society fragments.

The fragmented phase with most of the triads balanced agrees with the result of^47^. However, a crucial difference between the two models is that increasing *G* leads to a balanced society in^47^, but to the destruction of social balance in ours. While the reason for this difference is not fully clear to us, it seems that the probabilities of link updates in the two models depend on *G* in different ways. While less link updates can happen as *G* increases in our model, a large *G* seems to retain the constant high activity of link updates in^47^ due to their local instant updating rule. Nevertheless, for small *Q*, the unbalanced steady states of both models have the same stationary values in the network observables. Note that if a term, equivalent to that of the *p* term, is introduced to Eq. (1), e.g. of the form $h\big (\sum _{(i,j)}(1-J_{ij})\big )$ for some external field strength *h*, then the “paradise state” can be reached for sufficiently large *h*, see^40^. Beyond a value $Q_{MF}$, the model produces very similar result to that obtained by minimising the Hamiltonian in Eq. (4)^40^. The existence of $Q_{MF}$ suggests that if individuals keep a large fraction of their local triads balanced then locally minimising an individual stress can become equivalent to reducing an overall stress. In comparison with these two approaches, the model presented here shows the possibility of social fragmentation being fully consistent with both, homophily and social balance theory.

The model, however, has a number of limitations that may be interesting to address in future work. The first is the choice of binary symmetric interaction coupling, $J_{ij} \in \{-1, 1\}$, which does not capture the possibility of non-reciprocal and weighted links, as well as the existence of higher-order interactions between individuals. Further, the actual relations between agents can be poorly estimated by such binary definition. For instance, people who are only extreme about one particular issue can become enemies despite of their similar opinions on all other topics. The second limitation comes from the use of unrealistic networks with a fixed ring topology. While this special case is chosen to highlight the key effect of balancing a sufficiently large number of triads on social balance of the entire network, more general cases with heterogeneity and/or adaptive changes in the topology, such as link rewiring, may not ensure such effect to happen. Nevertheless, since our model becomes equivalent to that of^40^ in the limit of $Q \rightarrow N^{\Delta }$, which does exhibit a balanced phase on both, time-varying topology and small-world topology, we conjecture that the main result of our model will still hold true for these topologies.

In the current implementation of the model all agents have the same fraction $Q / N^{\Delta }$. If $Q_i / N^{\Delta }$ varies from one individual to another, then, for some agents *i*, $Q_i / N^{\Delta }$ may become smaller than $q_{c}$ that is required to make all their $N_i^{\Delta }$ triads balanced. As a consequence, the whole network appears to be partly—but not perfectly—balanced (indeed $f \simeq 0.69-0.88$ in online societies^16,17^). Finally, one can generalise our treatment to the case of *interdependent* and *continuous* opinions on correlated topics, where, interestingly, an emergence of polarised ideological opinions has been observed^54^.

## Supplementary Information

Supplementary Information.
