# Supplementary material for: Balance and fragmentation in societies with homophily and social balance
Source: Sci Rep. 2021 Aug 25;11:17188. doi: 10.1038/s41598-021-96065-5 (PMC8387482; doi:10.1038/s41598-021-96065-5)
Supplement: Supplementary file 1 — Supplementary Information. [file 41598_2021_96065_MOESM1_ESM.pdf]

Supplementary Material to "Balance and fragmentation in societies with homophily and social balance"

Tuan M. Pham[1,2], **Andrew C. Alexander**[3], **Jan Korbel**[1,2], **Rudolf Hanel**[1,2], **and Stefan Thurner**[1,2,4,*]

[1]Section for the Science of Complex Systems, CeMSIIS, Medical University of Vienna, Spitalgasse 23, Vienna, A-1090, Austria
[2]Complexity Science Hub, Vienna Josefstädterstrasse 39, Vienna, A-1090, Austria
[3]Department of Mathematics, Princeton University, Princeton, NJ 08544, USA
[4]Santa Fe Institute, 1399 Hyde Park Road, Santa Fe, NM 87501, USA
[*]stefan.thurner@meduniwien.ac.at

1 The steady state of the stress dynamics in the limit $Q = 1$, $G \to \infty$ for a fully-connected network

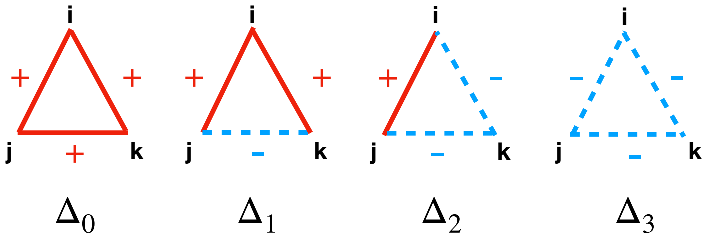

SM Fig. 1. Four different types of triads: Δ_k, with $k = 0,1,2,3$ being the number of negative links. In the limit $G \to \infty$, it is very unlikely, for example, that two positive links of the Δ_1 triad attached to i will change their signs at the same time if one of i's attribute flips, resulting in a Δ_3 triad.

When only one triad ($Q = 1$) containing node i is picked in an update, two links incident to i, say J_{ij} and J_{ik}, are to be redefined according to $J_{ij} = \mathrm{sign}(\mathbf{A}_i \cdot \mathbf{A}_j)$ and $J_{ik} = \mathrm{sign}(\mathbf{A}_i \cdot \mathbf{A}_k)$. From this definition, a change in the link J_{ij} from positive to negative can happen if, and only if, before the update there are $(G+1)/2$ common attributes between i and j and then one among them is selected to flip. This may happen with probability a_+. We can estimate a_+ by the following argument: As each agent i is endowed with G binary attributes, the number of i's possible states is 2^G. Now consider a neighbour j of i who currently has a positive link with i. This means that i can have any of the 2^{G-1} possible states compatible with that of j (the other 2^{G-1} states of i would result in a negative link between i and j). The probability a_+ of switching J_{ij} from positive to negative is given by the product of p_1 and p_2, where p_1 is the probability of picking a state of i out of the set of all compatible states which has exactly $(G+1)/2$ attributes similar to j before i flips one of its opinions and p_2 is the probability of uniformly selecting one similar attribute among all G attributes. This yields

$$a_+ = \underbrace{\frac{1}{2^{G-1}}\binom{G}{(G+1)/2}}_{p_1} \times \underbrace{\frac{G+1}{2G}}_{p_2},$$

The same argument applies for the probability a_- of switching a previously negative link to positive. Therefore, $a_- = a_+$. This reflects the fact that the process of a link flip is unbiased in our implementation. Note that

$$a_\pm \propto \frac{1}{\sqrt{G}}, \quad G \to \infty.$$

Let ρ_+ be the fraction of positive links. If one assumes that the link J_{ij} can be positive with probability ρ_+ or negative with probability $1-\rho_+$, then the probabilities that it changes from $+$ to $-$ and from $-$ to $+$, $\pi^{(+\to-)}$ and $\pi^{(-\to+)}$, respectively, are given by

$$\pi^{(+\to-)} = \rho_+ a_+ \,, \quad \pi^{(-\to+)} = (1-\rho_+) a_-$$

Among the three possible cases – namely, exactly 0, 1, or 2 of the links change sign – in the limit $G \to \infty$, we can neglect the case where both of them change at the same time as this scenario happens with vanishing probability. This means that in our treatment we consider only those update events during which at most one link can switch.

We formulate the set of differential equations describing the time evolution of triads in a fully-connected network, following Antal et al.[1]. Let Δ_k be as defined in SM Fig. 1. The corresponding density of Δ_k triads is defined by $n_k = N_k/M$, where N_k is the total number of triads of type Δ_k and $M \equiv \sum_k N_k = \binom{N}{3}$ is the total number of triads in a fully-connected network. We first define two variables: the density of triads of type Δ_k that are attached to a positive link, n_k^+,

$$n_k^+ = \frac{(3-k)n_k}{3n_0 + 2n_1 + n_2} \,,$$

and the density of triads of type Δ_k that are attached to a negative link, n_k^-,

$$n_k^- = \frac{k n_k}{n_1 + 2n_2 + 3n_3} \,.$$

Using n_k^+ and n_k^-, the set of ODEs can be written as

$$\begin{aligned}
\dot{n}_0 &= \pi^{(-\to+)} n_1^- - \pi^{(+\to-)} n_0^+ \\
\dot{n}_1 &= \pi^{(+\to-)} n_0^+ + \pi^{(-\to+)} n_2^- - \pi^{(-\to+)} n_1^- - \pi^{(+\to-)} n_1^+ \\
\dot{n}_2 &= \pi^{(+\to-)} n_1^+ + \pi^{(-\to+)} n_3^- - \pi^{(-\to+)} n_2^- - \pi^{(+\to-)} n_2^+ \\
\dot{n}_3 &= \pi^{(+\to-)} n_2^+ - \pi^{(-\to+)} n_3^- \,.
\end{aligned} \tag{1}$$

The stationary state is defined by setting the left sides of Eqs. (1) to zero and requiring $\pi^{(+\to-)} = \pi^{(-\to+)}$ for a fixed density of positive link. This implies

$$\begin{aligned}
& n_0^+ = n_1^- \,, \quad n_1^+ = n_2^- \,, \quad n_2^+ = n_3^- \\
& \rho_+^{(\mathrm{st})} a_+ = \big(1 - \rho_+^{(\mathrm{st})}\big) a_- \,.
\end{aligned} \tag{2}$$

As within our approximation scheme, $a_+ = a_-$, the steady state solution to Eqs. (2) is $\rho_+^{(\mathrm{st})} = 1/2$. A consequence of this is the density of triangles type Δ_k is given by $n_k = \binom{3}{k}/8$, resulting in $f^{(\mathrm{st})} = 0$ in the steady state. These values of $\rho_+^{(\mathrm{st})}$ and $f^{(\mathrm{st})}$ agrees with the finding in[2] for the case $G = O(N^\gamma)$, $N \to \infty$ with $\gamma < 2$ and for arbitrary p, where p is the tendency towards friendship in their paper. One would need to introduce a p dependence to the expression of a_+ and a_- to recover the (p-dependent) balanced phase of the phase diagram reported there.

2 Comparison with Gorski et. al. model

In this section we briefly comment on the similarities and the differences between our model and the model of[2] (the PRL from hereafter). First of all, both models aim at incorporating the effect of homophily and Heider's balance on social dynamics in coherent frameworks. The specifications of the two models, however, are different as listed below:

1. The PRL studies a stochastic process with a parameter p representing a desire to reach consensus. Our model, instead, considers an optimisation of the social stress in Eq. (1) with a parameter Q representing the number of (temporally changing) relevant triads in the neighbourhood of an individual.

2. The social networks in the PRL are fully-connected, while those in our model are regularly sparse. This difference in the network structures results in significantly different numbers of triangles which may change when an opinion is flipped. More precisely, whenever an unbalanced triad is chosen, then $N-2$ triangles are to be updated in the PRL, but only Q triangles are relevant to the dynamics of our model. This implies that in the thermodynamic limit an infinite number of triads is going to change in the PRL.

3. Our restriction to the Q relevant triads leads to a delayed update in the links' values compared to the simultaneous update in the PRL, where all links incident to an agent i are changed immediately once he flips his opinion. In our implementation, for those neighbours j of i who do not belong to any of these Q triangles, their links with i remain unchanged during the network update.

4. The symmetry between positive and negative links is maintained in our model (positive links can switch to negative, and vice versa, with the same probability). The PRL, apart from the case $p = 1/3$, generally considers the case in which this symmetry is broken ($p \neq 1/3$).

As a consequence of the aforementioned differences, the two models deliver rather different results:

1. For fixed values of Q, increasing the number of attributes G destroys the social balance in our model. In contrast, in the PRL *balanced* (either fragmented or paradise) states were showed to exist in an infinite system of $N \to \infty$ individuals with $G = O(N^\gamma)$ as long as $\gamma \geq 2$ (for any value of p).

2. For fixed values of G, in the limit of large Q only fragmented (not paradise) states are observed in our model. The transition from the fragmented phase to the paradise which occurs upon increasing p beyond 0.5 in the PRL, does not happen in our model, where the density of positive links is always $\rho_+ \simeq 0.5$.

3. In the limit of $G \to \infty$ and $Q = 1$ the steady state of our model is *unbalanced* regardless of how G scales with N. In the PRL, however, unbalanced states depend on the scaling of G with N – namely they can only exist in the thermodynamic limit for $G = O(N^\gamma)$ with $\gamma < 2$. Nevertheless, the two models have the same density of positive links $\rho_+ \simeq 0.5$ and the order parameter $f \simeq 0$ in the unbalanced phase.

3 Positive clusters in the fragmented phase

Here we consider a weaker form of clusters – the so-called "positive clusters". By a positive cluster we mean a set of nodes such that for any pair of members in this set there exists a path that consists of only positive links. We run the dynamics on a triangular lattice for $N = 100$ with free boundary conditions to check whether "positive clusters" can be formed. To this end, we choose only $Q = 2$ triads per individual in each update. In SM Fig. 2 we show a snapshot of the signed social network after reaching a stationary state. For a small value of $G = 3$, the formation of several positive clusters is clearly seen. Negative links are shown in light blue. This configuration belongs to the *fragmented phase* that shows a high degree of balance, $f \sim 0.9$. For more attributes, $G = 99$, a positive single cluster emerges that percolates the lattice. This is a realization in the *cohesive phase* that is clearly not balanced, and indeed $f \sim 0$.

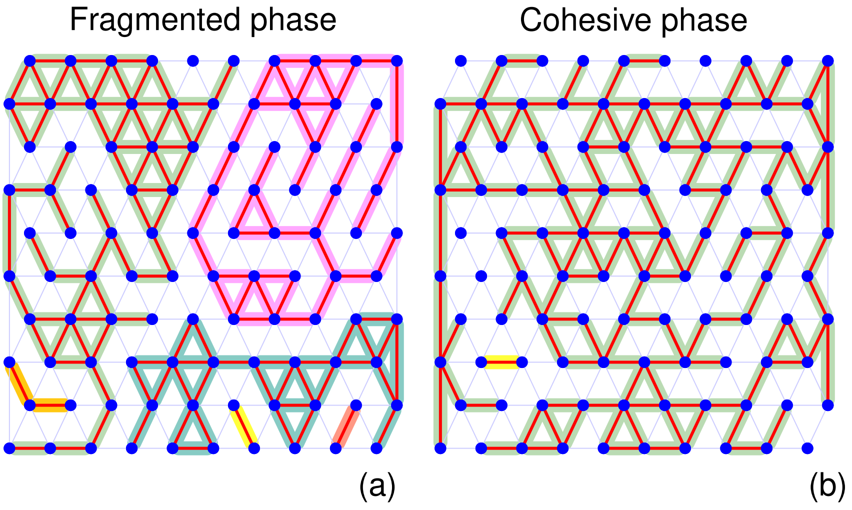

SM Fig. 2. (a) Positive clusters are identified in the fragmented and balanced phase, characterized by $G = 3$ opinions, while in (b) a single cluster appears in the cohesive phase for $G = 99$. $N = 100$, $Q = 2$. Note that $N^\Delta = 2, 3$, or 6, depending on the position of the agents on the lattice (free boundary conditions). Clusters are highlighted with colors.

4 Effect of the first term in Eq. (1)

Here we check what happens to the dynamics if the first term in Eq. (1) is switched off. We find in our simulation of finite size systems without the homophily term that balanced states can still be reached due to large fluctuations in the active phase. These fluctuations, however, become smaller as $N \to \infty$, resulting in a unbalanced quasi-stationary situation. The balanced state hence may not be observed within finite time. This shows that the effects of both terms are necessary for the convergence towards the balanced state (the sufficient condition is, of course, passing the critical value Q_c).

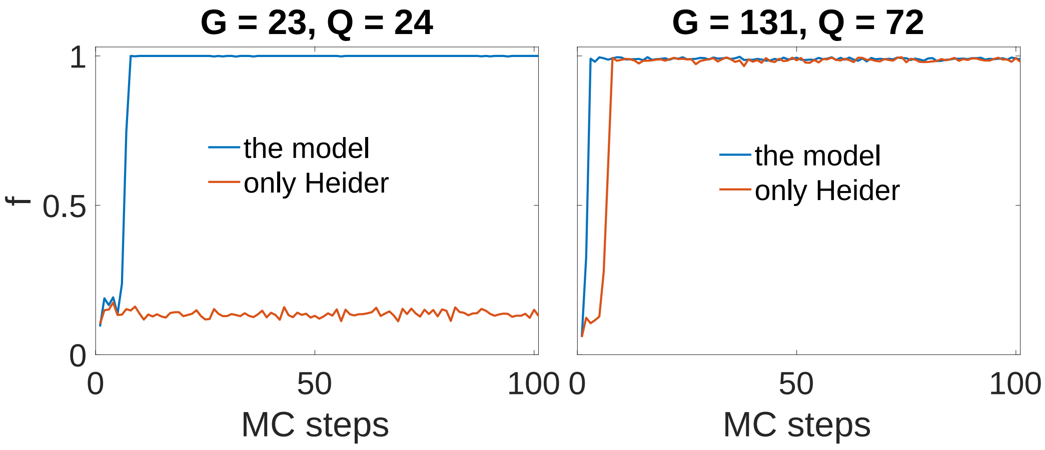

SM Fig. 3. Timeseries of f for a finite-size system of $N = 200$, $G = 23, Q = 24$ (left); and $G = 131, Q = 72$ (right) observed in the model simulation on networks with $K = 32$. Here one Monte Carlo step consists of $(N * G) \times (N * K)/2$ opinion flips.

5 Discontinuous transitions and bi-stability region

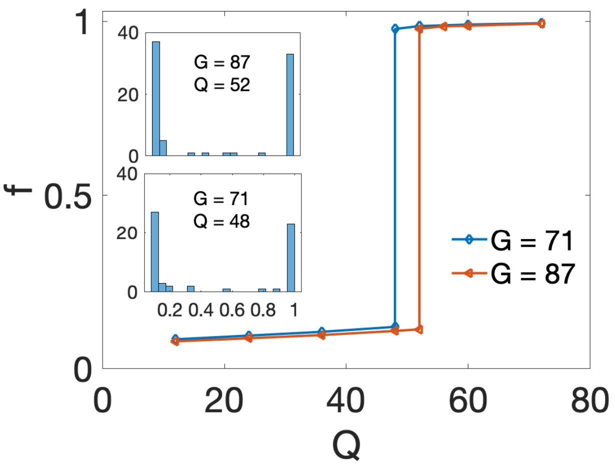

SM Fig. 4. Discontinuity in the transition at the bifurcation point. Here results are averaged over 100 simulations for $N = 400$, $K = 32$, $G = 87, Q = 52$ and $G = 71, Q = 48$. The insets show the corresponding histograms of f.

For fixed values of G the transition from cohesive to fragmented phase is discontinuous. We also observe that the distribution of the order parameter f becomes bimodal at the transition point, where f can be $f \simeq 0$ or $f \simeq 1$, indicating a metastable co-existence phase which may last for a rather long period of time until fluctuations bring the system to the absorbing states. Examples of this phenomenon are shown in SM Figure 4.

6 Details of the co-evolution based on the Hamiltonian in Eq. (4)

Starting from a random configuration of opinions and links, the society is updated from one timestep t to the next as follows:

1. Compute $\bar{H}$ of the current state of the system, assume it has a value of $\bar{H}_0$.

2. Pick a node i at random and flip one of its opinions, a_i^ℓ. Compute $\bar{H}$ again, it is now $\bar{H}_1$. If the value of $\bar{H}$ has decreased in response to the flip, $\bar{H}_1 \leq \bar{H}_0$, accept the flip. If the value of $\bar{H}$ increased, accept the flip only with probability $p = e^{-\Delta\bar{H}}$, where $\Delta\bar{H} = \bar{H}_1 - \bar{H}_0$ is the difference of stress before and after the flip. Pick the next node randomly and continue until N opinion updates have been performed.

3. Compute $\bar{H}$ of the system at this point, assume that it is now $\tilde{H}_0$. We now pick one link randomly, J_{ij}, and flip it. Compute $\bar{H}$ again, and assuming it to be $\tilde{H}_1$, we accept the flip if $\tilde{H}_1 \leq \tilde{H}_0$, and accept it with probability $p' = e^{-\Delta\tilde{H}}$, where $\Delta\tilde{H} = \tilde{H}_1 - \tilde{H}_0$, if $\tilde{H}_1 > \tilde{H}_0$.

4. Continue with the next timestep by returning to step 1.

7 Co-evolution based on the Hamiltonian in Eq. (4) with links determined by the opinions

One may ask what would happen to the co-evolutionary dynamics described in Eq. (4) if we update links after every opinion flip according to $J_{ij} = \text{sign}(\mathbf{A}_i \cdot \mathbf{A}_j)$ in step 3. This means that links are no longer random variables, but strictly determined by agent opinions. Still, those proposed changes that decrease the Hamiltonian in Eq. (4) are favoured over those that increase it, the same as before. Such a modified dynamics of Eq. (4) is rather similar to the presented model. Indeed, this variant can also be obtained if the same updating rule of the presented model with $Q = N^\Delta$ and the stress in Eq. (4) are used instead of that in Eq. (1). SM Figure 5 shows the phase diagram of this variant of Eq. (4) (a) and its section at various values of g (b) obtained for a fully-connected network. A sharp transition between the cohesive and fragmented phases is also observed. The result suggests further research on a possible correspondence between the Hamiltonian and our agent-based approach if the effect of social balance is sufficiently large.

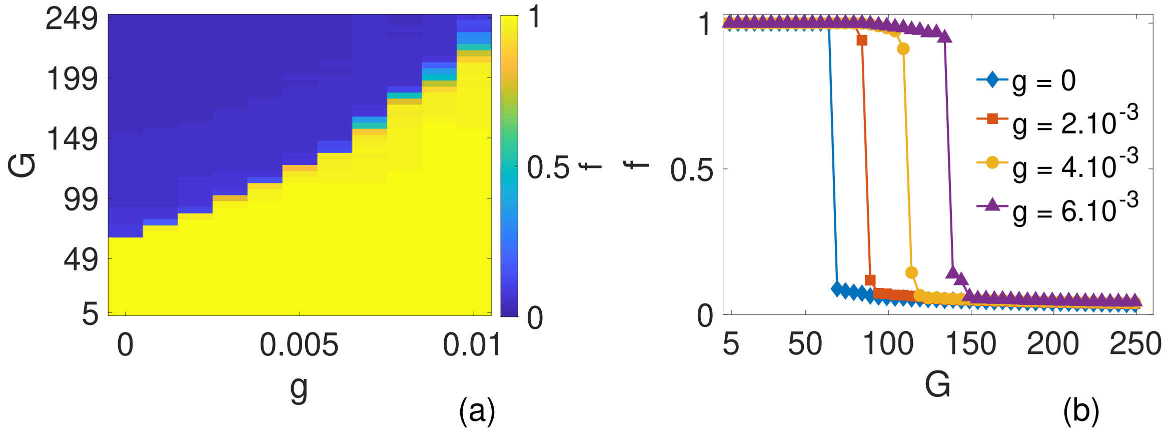

SM Fig. 5. Result of the model variant of Eq. (1) with strictly enforcing $J_{ij} = \text{sign}(\mathbf{A}_i \cdot \mathbf{A}_j)$. (a) f is shown as a function of g and G. (b) Section of the phase diagram for various strength of the coupling constant, g. Results are averaged over 100 runs for fully-connected networks of $N = 100$.

References

1. Antal, T., Krapivsky, P. L. & Redner, S. Dynamics of social balance on networks. *Phys. Rev. E* **72**, 036121 (2005).

2. Górski, P. J., Bochenina, K., Hołyst, J. A. & D'Souza, R. M. Homophily based on few attributes can impede structural balance. *Phys. Rev. Lett.* **125**, 078302 (2020).
